# MicroRNAs and Other Non-Coding RNAs as Regulators, Biomarkers, and Therapeutic Targets

**DOI:** 10.3390/ijms25073998

**Published:** 2024-04-03

**Authors:** Wan Lee

**Affiliations:** 1 Department of Biochemistry, Dongguk University College of Medicine, 123 Dongdae-ro, Gyeongju 38066, Republic of Korea; wanlee@dongguk.ac.kr; Tel.: +82-54-770-2409; Fax: +82-54-770-2447; 2 Section of Molecular and Cellular Medicine, Medical Institute of Dongguk University College of Medicine, 123 Dongdae-ro, Gyeongju 38066, Republic of Korea; 3 Channelopathy Research Center (CRC), Dongguk University College of Medicine, 32 Dongguk-ro, Ilsan Dong-gu, Goyang 10326, Republic of Korea

## 1. Introduction

Over the past few decades, the field of RNA research in molecular and cellular biology has undergone a dramatic transformation. In particular, the study of non-coding RNAs (ncRNAs) has become the center of major academic activities, moving from being the focus of minor genetic research. MicroRNAs (miRNAs), long non-coding RNAs (lncRNAs), and circular RNAs (circRNAs) are examples of such molecules that do not encode proteins but are crucial regulators of gene expression. Their essential functions range from orchestrating the epigenetic modification of gene expression to arranging subtle intercellular communication, making them vital custodians in a number of physiological conditions and diseases. Growing recognition of the roles of ncRNAs in human health and cellular mechanisms has prompted efforts to investigate their regulation and implications in health and diseases.

The expansive nature of ncRNA research now encompasses a holistic approach, considering the intricate network of interactions that govern cellular function. Innovations in sequencing technologies and bioinformatics tools have enabled a deeper understanding of the ncRNA transcriptome, revealing a more complex landscape. The discovery of myriad ncRNA species, each with distinct functions and mechanisms of action, underscores the versatility of these molecules in regulating gene expression. The intricate dance of ncRNAs within the cellular milieu reveals a regulatory network of staggering complexity, where ncRNAs act not merely as molecular switches but also as orchestrators of cellular fate and function. This realization has propelled the field into new territories, exploring the synergistic interactions between ncRNAs and other biomolecules and their collective impact on cellular architecture and dynamics.

The convergence of ncRNA research with emerging scientific paradigms is not only expanding our knowledge horizon, but it is also challenging existing models of genetic regulation and disease pathogenesis. In recent years, studies have shed light on the critical roles played by ncRNAs in a variety of human diseases, including cancer, neurological disorders, and metabolic disorders. These studies have mapped out promising pathways for ncRNAs as diagnostic pre-cursors, therapeutic designers, and prognostic predictors. These trials have paved the way for developing ncRNA-based diagnostic tools and therapeutics, offering hope for personalized medicine tailored to the molecular underpinnings of individual diseases. Given their flexibility and selectivity in regulating gene expression, ncRNAs are attractive therapeutic targets. Researchers are utilizing the unique characteristics of ncRNAs to develop RNA-based treatments that may revolutionize the treatment of complex diseases by utilizing these treatments. Further investigation of ncRNAs has identified their role in maintaining cellular homeostasis and regulating gene expressions necessary for robustness. For advancing our understanding and uncovering new research avenues, cutting-edge technology and methodological advancements have been integral to ncRNA research.

This Special Issue will explore the most recent ncRNA research while showcasing the diverse roles and mechanisms of these molecules within various cellular and tissue environments. It highlights the trials conducted to elevate ncRNAs to innovative biomarkers and therapeutics, proving their immense potential for monitoring human diseases. The combination of these studies’ findings will not only highlight the importance of ncRNAs in molecular and cellular biology but also provide new insights into human health and diseases.

## 2. Highlights of This Special Issue

This Special Issue aims to illuminate the roles and significance of ncRNAs in the maintenance of cellular homeostasis, their potential as diagnostic and prognostic biomarkers, and their utility as therapeutic targets. The first article (contribution 1) by Suzuki et al. explores the impact of the lncRNA LINC00173 in lung adenocarcinoma. This comprehensive study delves into the molecular mechanisms by which LINC00173 influences tumor progression, especially its interaction with the SNAIL transcription factor and FHIT tumor suppressor gene. Using various experimental approaches and functional assays in lung adenocarcinoma cell lines, these researchers found that LINC00173 acts as a molecular sponge for SNAIL, and thus prevents SNAIL from downregulating FHIT, a known tumor suppressor, ultimately inhibiting cancer cell migration and invasion. This novel LINC00173/SNAIL/FHIT regulatory axis highlights the complex interplay between lncRNAs in cancer biology and suggests that LINC00173 might serve as a potential biomarker for lung adenocarcinoma prognosis or as a target for therapeutic intervention. The study further substantiates its findings by analyzing patient data and showing that higher levels of LINC00173 correlate with better survival outcomes, underscoring a potential role in lung adenocarcinoma suppression. This study opens new avenues to understanding the molecular mechanisms responsible for lung adenocarcinoma and emphasizes the significance of lncRNAs in cancer progression and treatment strategies. 

The second article (contribution 2) by Liu et al. investigates the role of 17β-hydroxysteroid dehydrogenase type 12 (HSD17B12) and miR-136 in the adipogenic differentiation of adipose-derived stromal vascular fractions (SVFs). Previous studies have highlighted the importance of HSD17B12 in adipogenesis, but the regulatory mechanism involving the targeting of HSD17B12 by miR-136 in ovine adipogenesis was not fully understood. This study aimed to clarify this mechanism using bioinformatics and a dual-luciferase reporter assay, which confirmed that HSD17B12 is a direct target of miR-136. The authors demonstrate that miR-136 promotes the proliferation of ovine SVFs but inhibits their adipogenic differentiation. In contrast, HSD17B12 suppressed the proliferation and facilitated the adipogenic differentiation of these cells, which suggests that miR-136 aids the proliferation and hampers the adipogenic differentiation of ovine SVFs by targeting HSD17B12. The results of this study provide valuable insights into the complex regulatory mechanisms of lipid deposition in sheep and suggest that manipulating miR-136 and HSD17B12 levels might influence the balance between proliferation and differentiation in adipose tissues. These findings lay a theoretical foundation for further research on the processes that regulate fat deposition and offer potential strategies for managing adipogenesis.

The third article (contribution 3) by Yang et al. focuses on the role of PC-3p-2869 (miR-PC-2869) in antler growth and its potential therapeutic applications in osteosarcoma and chondrosarcoma. The authors found that miR-PC-2869 is extensively expressed in various layers of antler tissues and that its overexpression suppressed the migration and proliferation of antler cartilage cells. In addition, the heterologous expression of miR-PC-2869 in the osteosarcoma cell lines MG63 and U2OS and the chondrosarcoma cell line SW1353 reduced cell proliferation and migration. A reporter library screening identified 18 genes targeted by miR-PC-2869 in humans, including CDK8, EEF1A1, and NTN1, which share conserved binding sites with red deer. Furthermore, miR-PC-2869 overexpression downregulated these genes in antler cartilage and cancer cell lines. In addition, the knockdown of CDK8, EEF1A1, or NTN1 in these cells mimicked the suppressive effects of miR-PC-2869 overexpression. The authors also reported that miR-PC-2869 regulates the expressions of cyclin D1 and c-myc, which are crucial cell cycle regulators, via CDK8, EEF1A1, and NTN1, thus revealing the roles and mechanism of miR-PC-2869 and underscoring its potential as a therapeutic target in bone cancer.

In the fourth article (contribution 4), Han et al. investigate the role of miR-146b-5p in the regulation of inflammation in lipopolysaccharide (LPS)-treated human dental pulp cells (hDPCs) and its potential therapeutic implications. The study demonstrates that miR-146b-5p is upregulated in response to LPS stimulation and the subsequent upregulations of pro-inflammatory cytokines in hDPCs. This upregulation was suppressed by an NF-κB inhibitor and a JAK1/2 inhibitor, indicating that miR-146b-5p expression is regulated via an NF-κB/IL6/STAT3 signaling cascade. Interestingly, the ectopic expression of miR-146b-5p in hDPCs decreased NF-κB p65 phosphorylation, downregulated the expressions of pro-inflammatory cytokines, and inhibited the expressions of crucial NF-κB signaling components, including interleukin-1 receptor-associated kinase 1 (IRAK1), tumor necrosis factor receptor-associated factor 6 (TRAF6), and REL-associated protein (RELA). Additionally, the authors reported that the expressions of rat miR-146b-5p (rno-miR-146b-5p) and pro-inflammatory cytokine mRNAs are upregulated in experimentally induced rat pulpal inflammation in vivo. Moreover, in LPS-stimulated ex vivo cultured rat incisor pulp tissues, rno-miR-146b-5p effectively blocked the mRNA expressions of pro-inflammatory mediators and NF-κB signaling components. These findings suggest that miR-146b-5p plays a crucial role in downregulating the expressions of pro-inflammatory mediators by targeting TRAF6, IRAK1, and RELA in LPS-stimulated hDPCs and highlight its potential use as a therapeutic target for controlling dental pulp inflammation.

The fifth article (contribution 5) by Abgoon et al. investigates the persistence of changes in serum microRNA (miRNA) levels in patients with sudden sensorineural hearing loss (SSNHL), which is characterized by idiopathic, rapid onset hearing loss. It had been previously demonstrated that specific miRNAs (miR-195-5p, miR-132-3p, miR-30a-3p, miR-128-3p, miR-140-3p, miR-186-5p, miR-375-3p, and miR-590-5p) exhibit altered expressions within 28 days of hearing loss onset in SSNHL. The authors aimed to determine whether these miRNA expressional changes persist in the long-term. They also compared the serum miRNA profiles of SSNHL patients within one month of hearing loss onset (the immediate group, *n* = 14 patients) with the profiles of patients 3 to 12 months post-onset (the delayed group, *n* = 9 patients). The groups were matched for age and sex, and expression levels of target miRNAs were measured using real-time PCR. In addition, both groups evaluated air conduction pure-tone-averaged (PTA) audiometric thresholds in affected ears at initial and final follow-up visits. No significant intergroup differences in miRNA expression levels, hearing recovery statuses and initial or final affected ear PTA audiometric thresholds were observed, which suggests that miRNA expression changes in SSNHL patients are stable over time and that no significant changes are evident at up to 12 months after hearing loss onset.

In the sixth study (contribution 6), Nguyen et al. explore the impact of miR-302a on the expression of twinfilin-1 (TWF1), actin filament modulation, cell proliferation, and myogenic differentiation in C2C12 progenitor cells. TWF1, an actin-depolymerizing factor, is crucially required for actin dynamics and myoblast differentiation. The study demonstrates that palmitic acid (PA), a common dietary saturated fatty acid (SFA), reduces TWF1 expression and hinders myogenic differentiation, while concurrently elevating miR-302a levels in C2C12 myoblasts. Intriguingly, miR-302a was found to directly suppress TWF1 expression by targeting its 3′ UTR, and the ectopic expression of miR-302a promoted cell cycle progression and proliferation by increasing filamentous actin (F-actin) accumulation, leading to the nuclear translocation of Yes-associated protein 1 (YAP1). Furthermore, this process suppressed key myogenic factors (MyoD, MyoG, and MyHC), thereby impairing myoblast differentiation. The authors concluded that SFA-inducible miR-302a epigenetically suppresses TWF1 expression and disrupts myogenic differentiation by promoting myoblast proliferation through an F-actin-mediated YAP1 activation pathway. This insight expands our understanding of the molecular mechanisms by which SFAs influence muscle differentiation and suggests a novel link between dietary components and myogenesis.

In the last article (contribution 7), Altman et al. comprehensively compile and analyze current research on the presence and implications of miRNAs in human tear fluid and highlight their associations with several ocular diseases, including dry eye disease, Sjögren’s syndrome, keratitis, vernal keratoconjunctivitis, glaucoma, diabetic macular edema, and diabetic retinopathy. In addition, this review was extended to non-ocular diseases, including Alzheimer’s and breast cancer, and thus, highlights the systemic relevance of tear fluid miRNAs. Furthermore, a summary of the known roles of these miRNAs provides development cues in this field and emphasizes the potential use of miRNAs in tear fluid as biomarkers for a range of diseases.

